# Solution of Gutta Percha in Chloroform

**Published:** 1851-10

**Authors:** 


*Solution of Gutta Percha in Chloroform.*—Dr. Rapp, of Bamberg, having observed that collodion, when applied for surgical purposes, loosens, if the skin is moist or transpiring, or if blood trickles down, has therefore, in place of it, employed a solution of gutta percha in chloroform, 7 grs. to ℥j., and speaks of it as forming a very pleasant and effectual adhesive dressing. A solution of one part to eight or nine, is as easily applied by a pencil as collodion.—*London Med. Times, from Buchner's Repert.*

